# Assessing Herbivorous Impacts of *Apohyale* sp. on the *Ulva prolifera* Green Tide in China

**DOI:** 10.3389/fpls.2021.795560

**Published:** 2021-12-15

**Authors:** Xiaoxiang Miao, Jie Xiao, Shiliang Fan, Yu Zang, Xuelei Zhang, Zongling Wang

**Affiliations:** ^1^College of Environmental Science and Engineering, Ocean University of China, Qingdao, China; ^2^Key Laboratory of Marine Eco-Environmental Science and Technology, First Institute of Oceanography, Ministry of Natural Resources, Qingdao, China; ^3^Laboratory of Marine Ecology and Environmental Science, Pilot National Laboratory for Marine Science and Technology (Qingdao), Qingdao, China

**Keywords:** *Ulva prolifera*, *Apohyale* sp., grazing, green tides, Yellow Sea

## Abstract

An epiphytic gammarid species, *Apohyale* sp., was abundant in the floating *Ulva prolifera* (*U. prolifera*), which forms large-scale green tides in the Yellow Sea (YSGT). Field observation and laboratory experiments were subsequently conducted to study the species identity, abundance, and grazing effects on the floating algal biomass. The abundance of *Apohyale* sp. showed great spatial variation and varied from 0.03 to 1.47 inds g^−1^ in the YSGT. In average, each gram of *Apohyale* sp. body mass can consume 0.43 and 0.60 g algal mass of *U. prolifera* per day, and the grazing rates varied among the algae cultured with different nutritional seawaters. It was estimated that grazing of *Apohyale* sp. could efficiently reduce ~0.4 and 16.6% of the algal growth rates in Rudong and Qingdao, respectively. The *U. prolifera* fragments resulting from gnawing of *Apohyale* sp. had a higher growth rate and similar photosynthetic activities compared to the floating algae, indicating probably positive feedback on the floating algal biomass. This research corroborated the significant impact of *Apohyale* sp. on the floating algal mass of YSGT through the top-down control. However, further research is needed to understand the population dynamics of these primary predators and hence their correlation with the expansion or decline of YSGT, especially under the complex food webs in the southern Yellow Sea.

## Introduction

**Figure 1:**
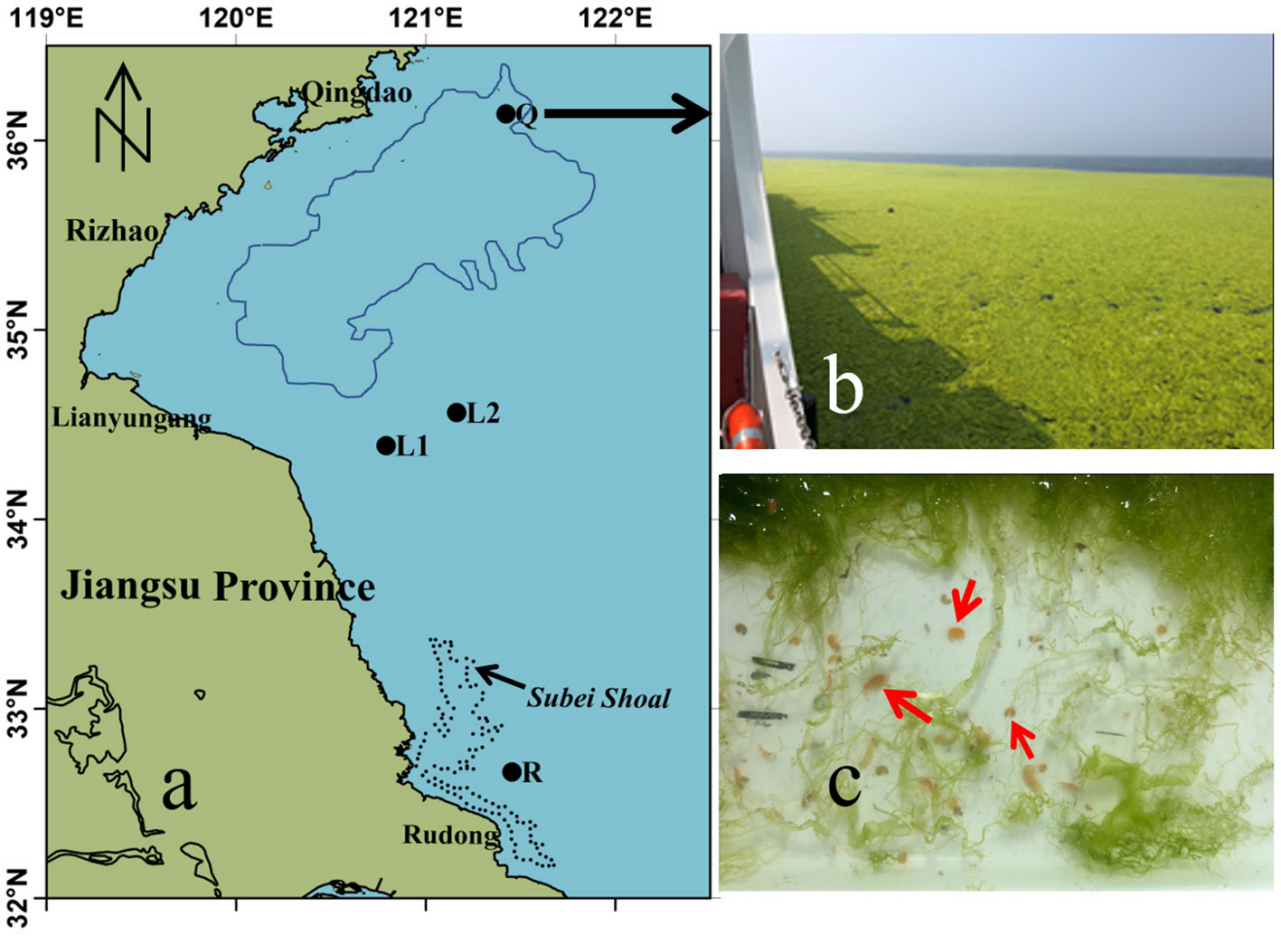
Maps of sampling stations in the southwestern Yellow Sea. **(a)** locations of sampling stations Q (Qingdao), L1 and L2 (Lianyungang) and R (Rudong, Subei Shoal). The blue line encloses the distribution of floating *U. prolifera* green tide on June 8, 2020 based on the satellite remote sensing, and the black dotted line is the region of *Pyropia* aquaculture in Subei Shoal. **(b)** Field photo of the floating algae at Station Q. **(c)** *Apohyale* sp. in the green macroalgae sample.

Green tides are ecological disasters caused by the aggregation of green macroalgae (Fletcher, 1996; Blomster et al., 2002), which are frequently occurring in coastal countries and regions around the world (Smetacek and Zingone, 2013). Since 2008, the large-scale *Ulva prolifera* (*U. prolifera*) green tides recur annually in the southern Yellow Sea of China and are recognized to be the largest green tide of the world (Liu et al., 2009; Yu and Liu, 2016). Various studies have confirmed a general raft-origin and northward drifting process of the Yellow Sea green tides (YSGT) and revealed a number of physiological and molecular mechanisms of *U. prolifera* for blooming as well (Liu et al., 2009; Gao et al., 2010; Qiao et al., 2011; Wang et al., 2015; Liu D. et al., 2020). Distinct from the numerous local green tides around the world, the YSGT undergoes long-distance drifting and causes significant trans-regional impacts with huge floating biomass inundating the open and coastal waters. The drifting biomass of *U. prolifera* originates from Subei Shoal, spreads rapidly throughout the southern Yellow Sea, and eventually reaches the south coast of Shandong Peninsula (Figure 1a). The free-floating *U. prolifera* explosively grows during drifting, forming the large-scale green tides (Huang et al., 2014; Bao et al., 2015). In general, consistent source biomass, eutrophication, suitable temperatures, seasonal monsoon, and wind-driven surface circulation in the southern Yellow Sea are the main factors stimulating and driving the blooms (Taylor et al., 2001; Morand and Merceron, 2004; Teichberg et al., 2010; Song et al., 2015). Little, however, is known about the roles of biotic factors, such as predators, on the occurrence of the green tides in the complex ecosystem of the southern Yellow Sea.

Gammarid-like animals have been commonly observed in the free-floating *U. prolifera* patches during multiple field surveys on YSGTs. They were present within the filamentous matrices of most drifting slicks of *U. prolifera* (Figure 1c). It remained unclear about the species, diversity, and functions of these epiphytic animals on the YSGTs. Gammarid is a species-rich taxon in the order Amphipoda of class Crustacea and accounted for 80% of the species in Amphipoda (Cruz-Rivera and Hay, 2000). They mainly inhabit the macroalgae fauna in intertidal zones, temperate estuaries, and enclosed or semi-enclosed waters, where they prey on macroalgae and/or organic detritus (Balducci et al., 2001; Jeong et al., 2007). Macroalgae provide sufficient food for gammarids and their dense canopy structure provides the ideal habitat (Duffy, 1990; Gestoso et al., 2013). On the other hand, predation of gammarids may control the biomass of algal primary producers (Duffy, 1990). A few species were reported associated with the drifting seaweed communities (*Sargassum* in particular), while their ecological roles were unclear (Sano et al., 2003). Even less was known about the distribution, abundance, and interactions of gammarid with the free-floating green seaweeds, especially the YSGT. Limited laboratory tests showed that certain gammarid species (*Eogammarus possjeticus*) had an obvious feeding preference on *U. prolifera*, suggesting a plausible containment measure on floating green seaweeds in closed or semi-closed mariculture ponds (Xue et al., 2018). Wang G. C. et al. (2020) noted the presence of gnawing gammarids in floating *U. prolifera* of YSGTs and speculated important ecological roles in regulating the floating *U. prolifera* biomass. Two different effects of gammarids were proposed: (1) the epiphytic gammarids decrease the biomass of free-floating *U. prolifera* by significant grazing and function as “top-down” control; and (2) gnawing of gammarids may induce sporangium of the algal fragments and lead to biomass increase. These hypotheses revealed likely discrepant ecological functions of gammarids on YSGTs, which are yet to be tested or verified by field data.

In this study, we focused on the epiphytic gammarids in the floating *U. prolifera* in the southern Yellow Sea, studied the abundance and species composition of the populations in the upstream source (Subei Shoal) and downstream aggregation regions (Qingdao) of the YSGT. Laboratory grazing experiment was also conducted to investigate the impacts of these gammarids on the biomass dynamics of floating *U. prolifera* and hence its ecological function on YSGT.

## Materials and Methods

### Sample Collection

Floating green macroalgae were sampled from the Subei Shoal off Rudong (R: 32°39′50″N, 121°27′14″N, Figure 1), Lianyungang coastal water (L1: 34°23′24″N, 120°47′24″N; L2: 34°33′47″N, 121°09′43″N), and Qingdao coastal water (Q: 36°08′13″N, 121°25′20″N) on May 22, 2019, June 4, 2020, and June 8, 2020, respectively. A WP2 zooplankton net with a mesh size of 200 μm and an inner opening diameter of 0.8 m was mounted on the side of the vessel, towed at three knots for 10 min through the surface water to collect the floating green macroalgae and epiphytic animals. The collected macroalgae were rinsed with sterilized seawater repeatedly. The washing fluid was then sieved (0.5 mm aperture) to collect the epiphytic animals. All animals retained by the sieve were counted. Selected animals were preserved in 5% formaldehyde or fixed in 95% EtOH for further species identification, and the rests were cultured with *U. prolifera* for the grazing experiments below. Green macroalgae were identified for species (Xiao et al., 2013) and weighed after being spun at 100 rpm for 3 min to remove excess water. The abundance of gammarids (*A*, inds g^−1^) was expressed as a function of green macroalgae wet mass.

### Identification of Gammarid Species

The gammarids were observed under the dissecting microscope. Morphological characters, namely, antenna, gnathopod, pereopods, uropods, and plates, were recorded by the microscope camera DS-Ri2 (Nikon, Japan) and compared with the references (Ren, 2006; Kilgallen, 2011; Lowry and Myers, 2013; Ratnasingham and Hebert, 2013). Body length was measured. Ten ethanol-fixed samples were randomly selected and rinsed with deionized (DI) water. Approximately 100 μg of muscle tissues from each sample was lysed and extracted for the genomic DNA using an E.Z.N.A.^TM^ tissue DNA kit (Omega Bio-Tek, Norcross, GA, USA). Mitochondrial cytochrome oxidase I (COI) fragment was amplified using the universal primer pair: LCO1490 5′-GGTCAACAAATCATAAAGATATTGG-3′ and HCO2198 5′-TTAACTTCAGGGTGACCAAAAAATCA-3′ (Folmer et al., 1994). PCR amplification program was as follows: an initial denaturation step at 95°C for 5 min, followed by 40 cycles of denaturing at 94°C for 40 s, annealing at 54°C for 40 s, extending at 72°C for 60 s, and finally extending at 72°C for 10 min before storing at 4°C. The PCR products were then analyzed by the electrophoresis on a 1% agarose gel stained with ethidium bromide and visualized under the Azure c150 image system (Azure Biosystem Inc., Dublin, CA USA). Selected products were purified using the E.Z.N.A.^TM^ cycling kit (Omega Bio-Tek, Norcross, GA, USA) and then sequenced bi-directionally by Sangon Biotech Co., Ltd. (China).

The obtained sequences were firstly basic local alignment search tool (BLAST) for the most similar sequences through the GenBank (http://www.ncbi.nlm.nih.gov). The reference sequences of 7 genera in Hyalidae were retrieved from the Barcode of Life Data System (Ratnasingham and Hebert, 2013) and then aligned with the sequence from this research by Mega version 6.0 (Tamura et al., 2013). Two sequences of *Hyalella azteca* from the neighbor family Hyalellidae were included as the outgroup. The redundant sequences of each genus were partially excluded to reduce the size of the dataset. The aligned sequences were transcribed into amino acids using the invertebrate mitochondrial genetic code to justify the alignment and nucleotide sequences. The phylogenetic trees of both amino acid and nucleotide sequences were constructed using Maximum-likelihood (ML) algorithms and the models with the lowest Bayesian Information Criterion (BIC) scores. The reliability of internal branches was evaluated with non-parametric bootstrapping for 1,000 replicates.

### Grazing Experiments

The grazing experiments were conducted in the Artificial Climatic Chamber (202728–380, Jiangnan Inc., Ningbo, China) at 16°C with 100 μmol photons m^−2^ s^−1^ light intensity and 12:12-h light:dark cycle. Ambient seawater was collected from both stations Rudong (R) and Qingdao (Q) to simulate the grazing effects under the natural nutritional and growing conditions of the floating algae. The surface seawater was filtered through the 0.22 μm cellulose acetate membrane and autoclaved before using it. Approximately 10 g fresh floating *U. prolifera* mass was weighted and cultured in a glass beaker with 1 L ambient seawater. The floating algae were rinsed with sterilized seawater multiple times to remove the epiphytic microalgae. Healthy and active gammarids were selected for the grazing experiment, and the density in each beaker was determined based on the natural abundance of the grazers in the field (see section Results below). For comparison, 15 gammarid animals (~225–300 mg body mass) collected from Station Q were added to each replicate of the grazing treatments R and Q. Four treatments (R, R′, Q, and Q′) with three replicates for each were set up to compare the variable growth rates of the floating *U. prolifera* and grazing effects of gammarids. Treatments R and R′ were cultured using the seawater from Rudong with (R) and without (R′) grazing animals, while Q and Q′ were seawater from Qingdao coast with (Q) or without (Q′) grazing animals. Seawater was refreshed every 2 days and the experiment lasted 19 d.

The *U. prolifera* mass and gammarid animals were weighted by the analytical balance every 2 days when refreshing the seawater. The relative growth rate (RGR) of *U. prolifera* and grazing rate of gammarids (GR) were calculated accordingly.


(1) $$RGR=100\times \frac{\ln W_{2}-lnW_{1}}{T_{2}-T_{1}}$$

where *RGR* is the relative growth rate (% d^−1^), *W*_1_ and *W*_2_ are the wet weight (g) of cultured algae at times *T*_1_ and *T*_2_, respectively.


(2) $$\begin{array}{ll} GR=\left[W_{1}•\left(\frac{W_{2}^{\prime }}{W_{1}^{\prime }}\right)-W_{2}\right]/[B•(T_{2}-T_{1})] & \end{array}$$

where GR is the grazing rate (g g^−1^ d^−1^) of gammarids, *W*_1_ and *W*_2_ are the algal mass (g) of the grazing treatments at *T*_1_ and *T*_2._ $W_{1}^{\prime }$ and $W_{2}^{\prime }$ are the algal mass (g) of the group without grazing. *B* is the mass weight (g) of gammarids in each culture.

To simulate the contribution of grazing on the loss of floating algal mass in blooming, we estimated the total algal biomass consumed by gammarids (grazing amount) in different regions with the variable density of gammarid and floating *U. prolifera* algae and further compared with the growth rate of floating *U. prolifera* biomass. The grazing amount (*G*, g d^−1^) was calculated:


(3) G=GR×A×B×m

where *A* is the abundance of gammarids (inds g^−1^) and *B* is the biomass of free-floating *U. prolifera* (g). The *m* is the average body weight of gammarids (g inds^−1^), which was 0.02 g inds^−1^ on average based on the measurement.

### Growth of *U. prolifera* Fragments

To test the growing potential of *U. prolifera* fragments resulting from gnawing of animals, algal fragments were collected from the bottom of beakers and gently transferred to the new beakers. About 2 g of algal fragments were weighed and cultured in each beaker with 200 ml ambient seawater. There were two treatments, culturing with the seawater from stations R and Q and three replicates for each. Seawater was renewed every 3 days, and the wet weight of algal fragments was recorded. Growth rates of cultured fragments were computed by Eq. (1).

### Nutrients of Seawater and Chlorophyll Fluorescence of Algae

To measure the nutrients, seawater was filtered through the 0.45 μm Waterman GF/F membrane and stored at −20°C in dark. Nutrients, such as DIN (NO_2_-N, NO_3_-N, and NH_4_-N) and PO_4_-P, were analyzed with an AutoAnalyzer (BRAN and LUEBBE AA3, Germany) (Zhu, 2006).

To evaluate the photosynthetic activity of the floating *U. prolifera* algae and fragments after gnawing, *in vivo* chlorophyll fluorescence measurements were conducted with Imaging-PAM (Walz, Germany). The thalli were acclimated in the dark for 20 min before measurements (Zhao et al., 2013). The induction curve was performed under a measuring light (ML) from a 620 nm light-emitting diode (LED) and actinic light of 100 μmol photons m^−2^ s^−1^ from LED arrays for 315 s. Then a saturating pulse (SP) of 0.6 s duration and 10,000 μmol photons m^−2^ s^−1^ were delivered to the thalli. Fv/Fm and Y(II) were evaluated accordingly. Fv/Fm represents the PSII maximum quantum yield (Johnson et al., 1993), while Y(II) is the effective quantum yield of PSII.

### Data Processing

Statistical analysis was carried out with the SPSS 16.0 statistical program (SPSS Inc., Chicago, IL, USA). One-way ANOVA was used to examine the differences in RGRs. Normality and variance homogeneity were analyzed by using the Shapiro–Wilk normality test and Levene's test for homogeneity of variances. Differences between abundance and GRs of gammarids among stations, nutrients of seawater, and chlorophyll fluorescence of algae were assessed *via* student *t*-tests. Normality assessment was also performed *via* the Shapiro–Wilk test. Measurement data were presented as the mean ± SE. The number (*n*, independent samples measured) for each analysis is given in the legends of figures. Significance levels for all tests were set at *p* < 0.05.

## Results

### Species Identification of Gammarids

**Figure 2:**
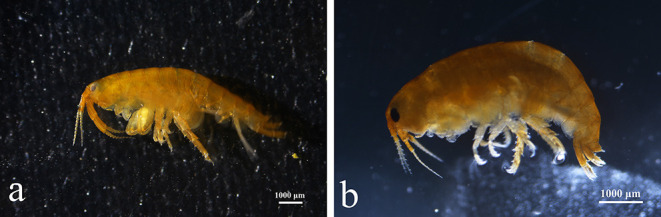
*Apohyale* sp. from station R **(a)** and Q **(b)**.

All the gammarid animals collected from the floating green algae shared a similar morphology with the family Hyalidae (Figure 2) (Lowry and Myers, 2013). Body length is from 0.8 to 1.3 cm. Eyes are large, oval, and black. There are two antennae, Antenna 1 is short and contains 12 segments, and Antenna 2 is slightly longer and contains 15 segments. Accessory flagellum for the antennae is absent. The second gnathopod is larger than the first. The propodus is rectangular with slanted edges. The third uropod is not segmented, and endopodite is minute or absent. The telsons are cleft. The main difference between *Apohyale* sp. and the closely related *A. pugettensis* is the shape of gnathopod. The gnathopod propodus of *Apohyale* sp. is rectangular with oblique edges, while that of *A. pugettensis* is trapezoid-like, and the notch was slightly bent (Ratnasingham and Hebert, 2013).

**Figure 3:**
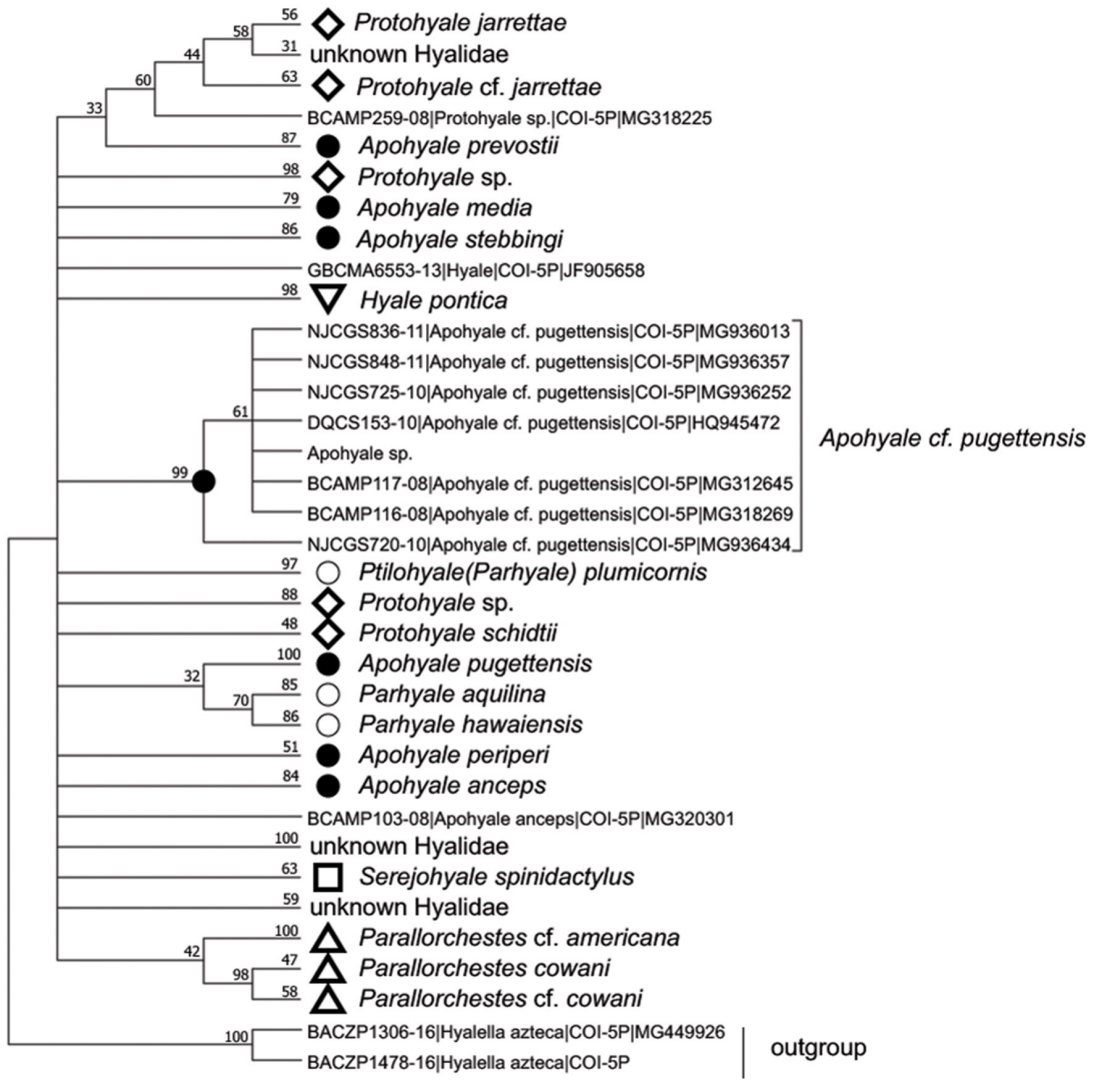
The condensed ML phylogenetic tree based on the amino acid sequences of COI in Hyalidae with cut-off value >30%. The monophyletic clade of each species was compressed and labeled with the specific markers and species names. Numbers above lines are bootstrapping support value (%) after 1,000 permutations. *Apohyale* sp. represents the sequence from this research. ML, Maximum-likelihood; COI, cytochrome oxidase I.

The COI sequences of 10 individuals were 100% identical, hence one representative sequence was used for the following analyses. BLAST search indicated that the sequence of *Apohyale* sp. was closest (91% similarity) to *A*. cf. *pugettensis*, a specimen identified on the northwestern coast of Canada. The sequence was aligned with 253 COI sequences from seven genera in Hyalidae. Phylogenetic analysis revealed multiple clades, which were mostly congruent with the species separation while informative at the genus level (Figure 3). The phylogenetic trees based on amino acids and nucleotides were quite similar. The sequences of each individual species (4 *Protohyale* spp., 3 *Parhyale* (*Ptilohyale*) spp., and 7 *Apohyale* spp.) formed well-supported monophyletic clades. Whereas, these three genera (*Protohyale, Parhyale*, and *Apohyale*) were paraphyletic and did not form reciprocal monophyletic clusters, suggesting probably unresolved phylogenetic relationships among the genera in this family. Combining with the morphological characters, we tentatively named the gammarid grazer detected in the floating *U. prolifera* of YSGT as *Apohyale* sp.

### Abundance of Gammarids in Floating Macroalgae

**Figure 4:**
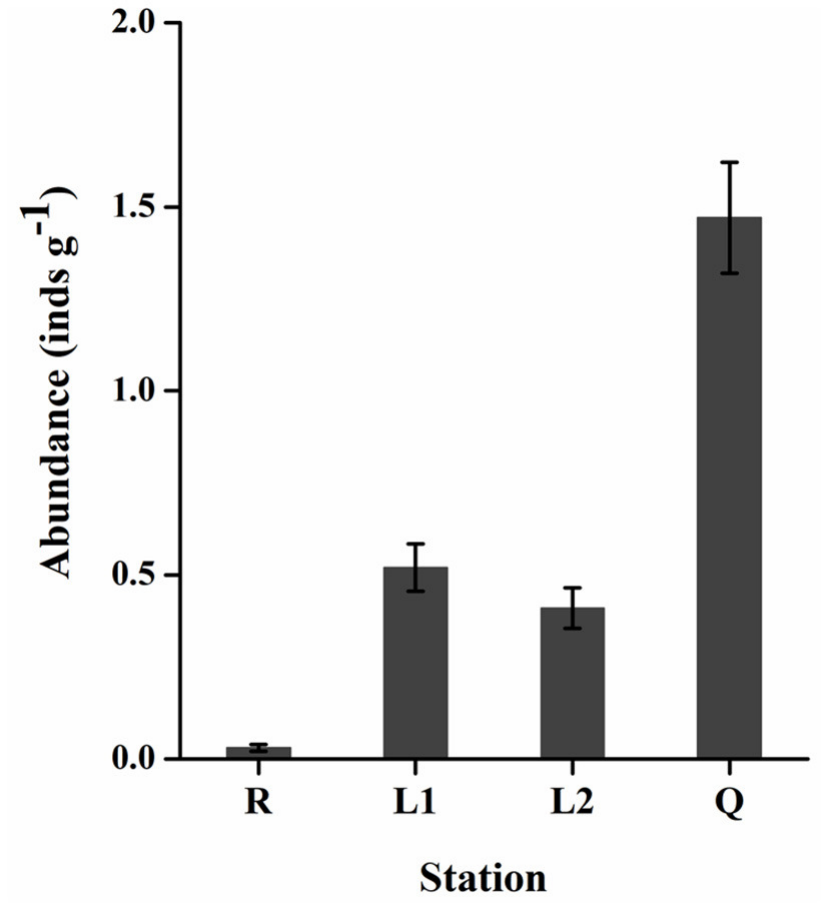
The abundance of *Apohyale* sp. in floating *U. prolifera* mats at 4 stations of YSGT. Error bars are SE (*n* = 3). YSGT, green tides in the Yellow Sea.

All the floating green macroalgae were identified as *U. prolifera* (data were not shown). As shown in Figure 4, the abundance of gammarids varied significantly (two-tailed student *t*-test, *p* < 0.05) between stations. There were only 0.02–0.05 (0.03 ± 0.01) gammarids per gram floating algae (inds g^−1^) at Rudong (R), while ~1.17–1.68 (1.47 ± 0.15) inds g^−1^ in the floating algae along the coast of Qingdao (Q). The abundance of gammarids in the floating algae along the coast of Lianyungang (L1, L2) was 0.45–0.65 (0.52 ± 0.07) inds g^−1^ and 0.34–0.52 (0.41 ± 0.05) inds g^−1^, respectively. The body length of the gammarid was 0.8–1.3 cm (1.0 cm on average), and no significant size differences were detected at different stations. Mating was commonly observed among the animals from station Q.

### Growth of Floating *U. prolifera* and Fragments

The growing curves of *U. prolifera* indicated significant influences from seawater used for culture and gammarid's grazing on the biomass of *U. prolifera*. Without grazing, the floating *U. prolifera* mass (R′ and Q′) grew rapidly during the first 15 days and then slowed down. The wet weight of Group R′ increased from 10.6 to 68.5 g within 19 d, and the RGR varied from 2.4 to 19.5% d^−1^ (10.4% d^−1^ on average). The algal biomass of Group Q′ was increased from 10.3 to 40.1 g within the culture period, and the RGR ranged from 0.3 to 12.9% d^−1^ (7.6% d^−1^ on average). The RGR of R′ (using seawater from Rudong) was evidently higher than Q′ (one-way ANOVA, *P* < 0.05), which could be explained by the higher nutrient level of seawater from Rudong (see below).

With grazing, the growth rates of *U. prolifera* biomass were significantly lowered. The algal biomass of Group R increased from 10.4 to 60.8 g after 19-d culturing, and the RGR was 2.2–17.6% d^−1^ (9.8% d^−1^ on average). Biomass of group Q was increased from 10.1 to 34.1 g (on average), and RGR was 1.9–12.4% d^−1^ (6.8% d^−1^ on average).

**Figure 5:**
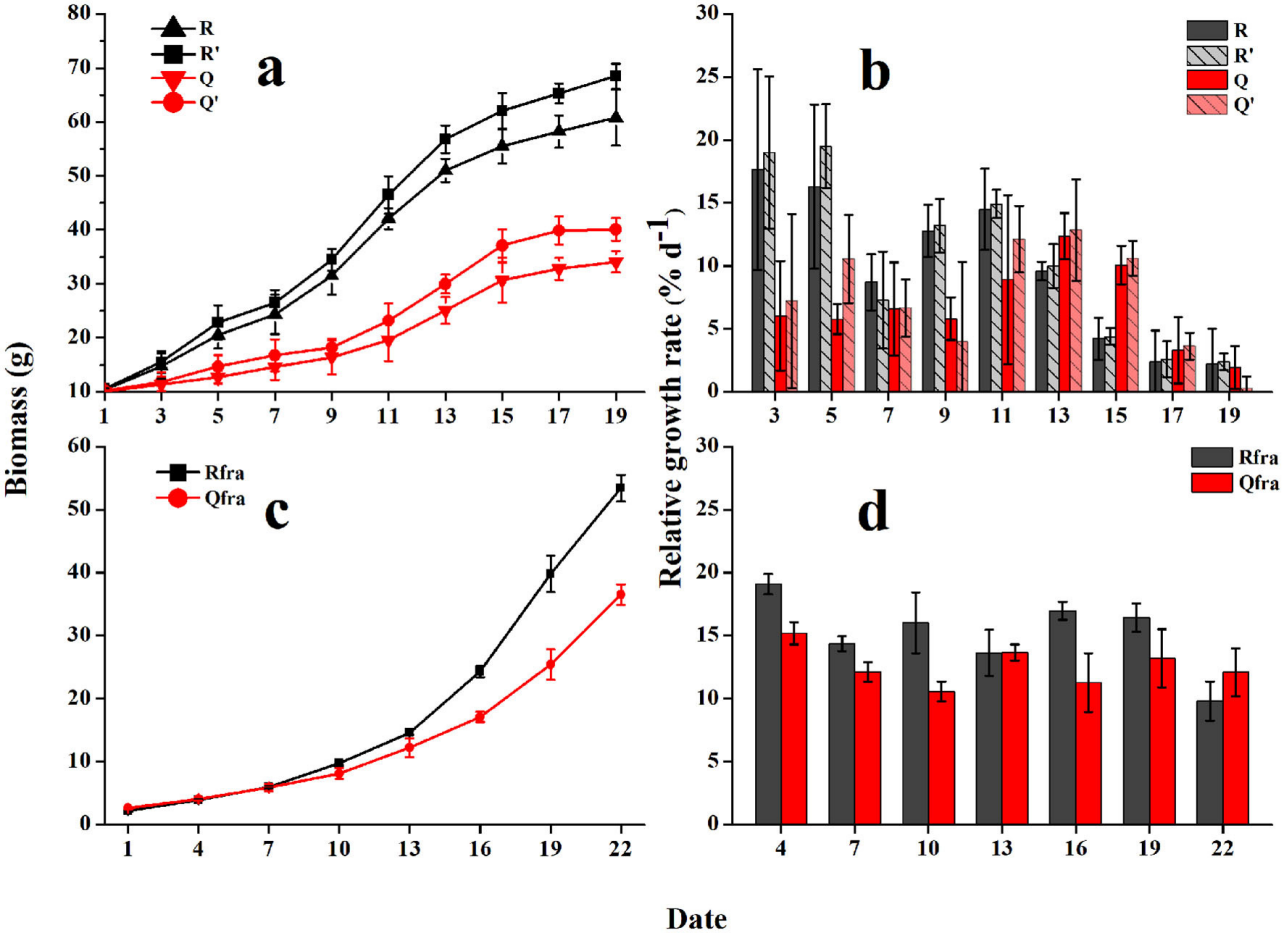
The growing curves **(a)** and relative growth rates [RGRs, **(b)**] of floating *U. prolifera* algae; and growth curves **(c)**, and RGRs **(d)** of fragments. Error bars are SE (*n* = 3).

The biomass of *U. prolifera* fragments grew exponentially, and the growth rates were obviously higher than those of floating *U. prolifera* algae (Figure 5). Similarly, different seawater for culturing could influence the growth rates of fragments. The fragment biomass in Group R was increased from 2.2 to 53.4 g (on average) within 22 days, and the mean *RGR* was 15.2% d^−1^. The biomass of Group Q was increased from 2.6 to 36.5 g, and the mean *RGR* was 12.6% d^−1^.

### Grazing Rate of Gammarid and Effect on the Biomass Loss of Floating Algae

**Figure 6:**
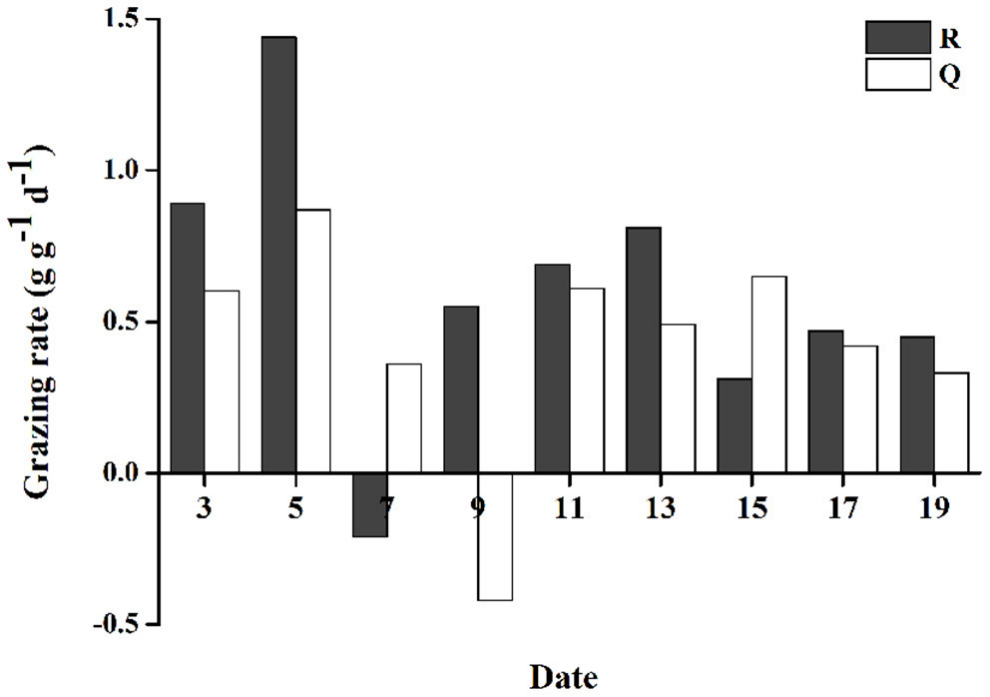
The grazing rate (GR) of *Apohyale* sp.

As described above, grazing of gammarids efficiently decreased the growth rates of floating *U. prolifera* mass. Although the floating *U. prolifera* mass with grazing animals (treatments R and Q) still grew steadily, the growth rates were obviously lower than the control groups (R' and Q', Figure 5). The GR varied from −0.21 to 1.44 g g^−1^ d^−1^ (0.60 g g^−1^ d^−1^ on average) for Group R, −0.42 to 0.87 g g^−1^ d^−1^ (0.43 g g^−1^ d^−1^ on average) for Group Q. The GRs of R were consistently higher than those of group Q (two-tailed student *t*-test, *p* < 0.05, Figure 6).

To evaluate the grazing effects on the floating algal biomass, we estimated the daily amount of algal mass consumed by the *Apohyale* sp. and compared it with the daily growing biomass of floating *U. prolifera*. As indicated in Table 1, the natural abundance of *Apohyale* sp. along the coasts of Qingdao (1.47 inds g^−1^) could lower the growth rate of floating *U. prolifera* by 16.6%. Whereas, *Apohyale* sp. in Rudong can only lower the algal growth rate by 0.4%, due to both the low abundance of *Apohyale* sp. and the high growth rate of *U. prolifera*.

**Table 1:** Simulating grazing effects of *Apohyale* sp. on the floating biomass of *U. prolifera*.

**Parameters**	***Apohyale* abundance (inds g^**−1**^) *A***	**Daily grazing (g)**	**Daily growing of floating *U. prolifera* (g)**	**Proportion (%)**
		**G = GR×A×B×m, (*m* = 0.02 g)**	**M = RGR×B**	**G/M×100**
QD	1.47	*G* = 0.0126 × *B*	*M* = 0.076 × *B*	16.6
*GR*_Apohyale_ = 0.43 g g^−1^ d^−1^;	2	*G* = 0.0172 × *B*		22.6
*RGR*_prolifera_ = 7.6% d^−1^	4	*G* = 0.0344 × *B*		45.3
	6	*G* = 0.0516 × *B*		67.9
	8	*G* = 0.0688 × *B*		90.5
	9	*G* = 0.0774 × *B*		101.8
RD	0.03	*G* = 0.0004 × *B*	*M* = 0.104 × *B*	0.4
*GR*_Apohyale_ = 0.60 g g^−1^ d^−1^;	2	*G* = 0.0240 × *B*		23.1
*RGR*_prolifera_ = 10.4% d^−1^	4	*G* = 0.0480 × *B*		46.2
	6	*G* = 0.0720 × *B*		69.2
	8	*G* = 0.0960 × *B*		92.3
	9	*G* = 0.1080 × *B*		103.8

Increasing the abundance of *Apohyale* sp. could potentially control or even prevent the biomass amplification of floating *U. prolifera*. For example, when the abundance of *Apohyale* sp. increased up to 8–9 inds g^−1^, the amount of algal mass consumed by *Apohyale* sp. could almost be equivalent to the growing amount of floating *U. prolifera* in coastal water of both Qingdao and Rudong (Table 1). Given the general bodyweight of *Apohyale* sp. *m* = 0.02 g ind^−1^, grazing of *Apohyale* sp. could prevent the biomass growth of floating *U. prolifera* when the mass weight of *Apohyale* sp. and *U. prolifera* was about 16:100–18:100.

### Photosynthetic Activities of Floating *U. prolifera* and Fragments

**Figure 7:**
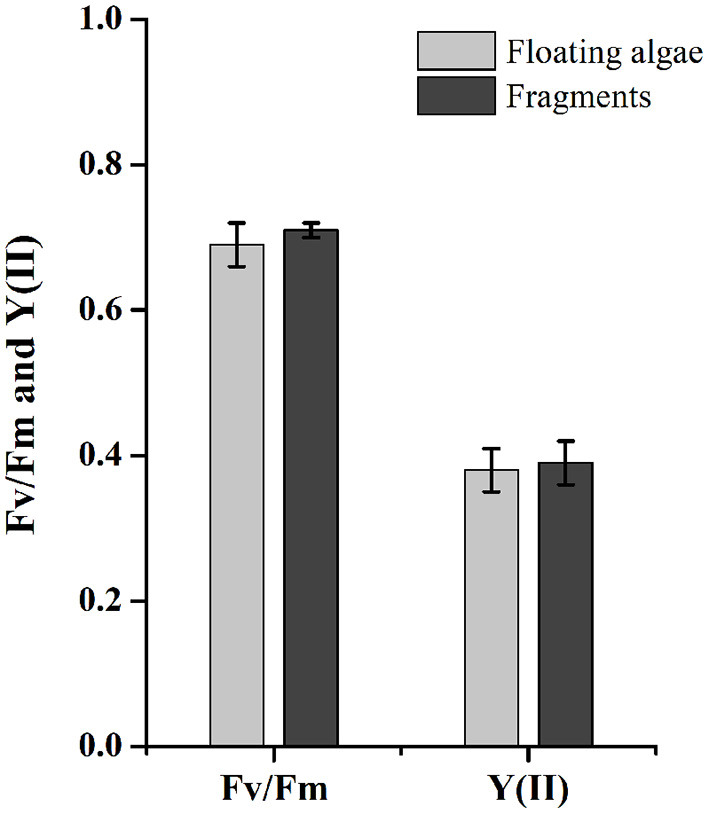
Fv/Fm and Y(II) of floating *U. prolifera* algae and fragments. Error bars are SE (*n* = 3).

There was no significant difference on Fv/Fm and Y(II) between floating clumps and fragmented *U. prolifera* (Figure 7). The Fv/Fm values of floating algae and fragments were 0.69 ± 0.03 and 0.71 ± 0.01, respectively (*P* = 0.133). The Y(II) were 0.38 ± 0.03 for floating algae and 0.39 ± 0.03 for fragments (*P* = 0.985). Gammarid grazing had no significant effect on the photosynthetic activity of *U. prolifera*.

### Nutrient Concentrations of Seawater

The nutrient concentrations of seawater from Station R were significantly higher than those from Station Q. As indicated in Table 2, the DIN and PO_4_-P concentrations at Station R were 16.42 and 0.12 μmol L^−1^, which were about 4 and 6 times the DIN (3.97 μmol L^−1^) and PO_4_-P concentration (0.02 μmol L^−1^) at Station Q, respectively.

**Table 2:** The nutrient concentrations (μmol L^−1^) of seawater.

**Nutrient**	***R***	***Q***
DIN	16.42	3.97
PO_4_-P	0.12	0.02
DIN/PO_4_-P	136.8	198.5

## Discussion

Herbivorous marine amphipods have long been recognized as important grazers on filamentous and ephemeral benthic algae (Duffy, 1990). The ecological roles of these herbivores diverse from trophic grazing, assisting the spore dispersal, to controlling the epiphytic microalgae and detritus, and hence regulating the species composition and fitness of the host benthic seaweeds across different ecosystems (Duffy, 1990; Sano et al., 2003; Valentine et al., 2006). With the increasing occurrence of floating macroalgal blooms in China's coastal waters (Qi et al., 2017; Liu et al., 2018; Song et al., 2019; Xiao et al., 2021), epiphytic amphipods have been noted and speculated to be important biological predators on the drifting seaweeds (Liu J. et al., 2020; Wang G. C. et al., 2020). Given the unique long-distance drifting and large geographic distribution of YSGTs, it is interesting to see whether the YSGTs could influence the populations of epiphytic grazers and *vice versa*. This study, for the first time, confirmed a specific gammarid species (*Apohyale* sp.) commonly existing and drifting with floating *U. prolifera* in the Yellow Sea. *Apohyale* sp. belongs to the Family Hyalidae, which is species-rich and cosmopolitan along the coasts around the world. Hyalidae has undergone a profound taxonomic change recently (Bousfield and Hendrycks, 2002). A number of sub-families and genera were revised and established with an emphasis on the North Pacific fauna, while the species diversity around the world remains unclear (Hiwatari et al., 2011; Kilgallen, 2011). There are in total over 200 species of 11–12 genera recorded (Desiderato et al., 2019; WoRMS Editorial Board, 2021), but only 37 species of 7 genera have sequences deposited (Ratnasingham and Hebert, 2013). Most of the sequences are from the coasts of the Atlantic, northeastern Pacific, and the coast of India (Ratnasingham and Hebert, 2013). Little is known about the genetic diversity of Hyalidae in the western Pacific, namely, China. Six morphological species in Hyalidae have been reported in Chinese seas, mostly found inhabiting intertidal and subtidal seaweeds in the tropical, subtropical, and temperate regions (Ren, 2006; Zheng et al., 2013). The phylogenetic status of *Apohyale* sp. was unresolved due to high species diversity while low coverage of sequence database and recent profound systematic changes in this family (Bousfield and Hendrycks, 2002; Best and Stachowicz, 2013; Iaciofano and Brutto, 2017; Desiderato et al., 2019). More research is needed to clarify its species identity, natural distribution, and habits.

The abundance of *Apohyale* sp. showed significant spatial variations. Based on our field observations, *Apohyale* sp. preferred shading habitat and tended to live within the matrices of filamentous macroalgal thalli. The low biomass and coverage of the floating *U. prolifera* in Rudong probably cannot provide enough shading for these animals. Another plausible explanation for this spatial variation was that active reproduction caused population expansion of *Apohyale* sp. in the floating mats during drifting. Mating was commonly observed in *Apohyale* sp. samples. Laboratory experiments indicated that it took about 35–40 days for juveniles to grow into mature adults, each gammarid female hatched once every 2–3 week and bred ~30 progenies (Xue, 2013). With this breeding rate and frequency, it was not surprising that the abundance of *Apohyale* sp. increased from 0.03 to 1.47 inds g^−1^ (about 40–50 times) during the 1-mo northward drifting with *U. prolifera* algae from Rudong to Qingdao coast. This demonstrates that macrobenthos can migrate with floating macroalgae, expanding their distribution and populations.

**Figure 8:**
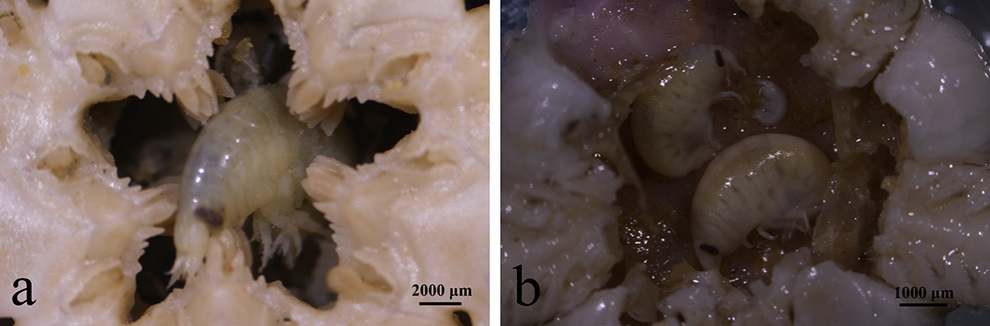
*Apohyale* sp. in gastric contents of *Stegophiura sladeni* at Station Q **(a,b)** sampled in June of 2020.

Besides the consistent source biomass from Subei Shoal, the total amount of floating biomass (or scale) of YSGTs was generally regulated by two pathways bottom-up and top-down controls. The former has been comprehensively studied, and the suitable environmental conditions, such as sufficient N nutrients and favorable temperature in the southern Yellow Sea, were determined to be the major factors boosting the floating biomass of YSGTs (Shi et al., 2015; Valiela et al., 2018; Zhang et al., 2020). Variable growth rates of *U. prolifera* could have resulted from the significant spatial variation of the nutrient levels along the coasts of Jiangsu and Shandong provinces (Chen et al., 2020; Wang C. et al., 2020). This was also confirmed by the current study as the *U. prolifera* cultured with seawater from Rudong had a higher growth rate. However, little was known about the impacts of top-down control on the floating biomass of *U. prolifera*. This study revealed an efficient GR of a dominant epiphytic gammarid (*Apohyale* sp.). Each gram of *Apohyale* body mass consumed ~0.43 and 0.60 g of *U. prolifera* algal mass every day. Given the natural abundances of *Apohyale* sp., such high grazing rates could reduce the growth rates of floating *U. prolifera* by 0.4 and 16.6% in the source and downstream regions (Rudong and Qingdao), respectively. Although this high grazing pressure cannot completely suppress the rapid growth of floating *U. prolifera* under the eutrophic environment, it was probably essential for the rapid decline of YSGTs at a late stage. Theoretically, increasing the grazers' abundance (up to 9 inds g^−1^, equivalent to 18:100 mass weight of *Apohyale* sp.: *U. prolifera*, Table 1) may turn over the increasing tendency of the floating *U. prolifera* biomass and hence prevent the expansion of the green tides. But the feasibility of this idealistic biological control method probably needs further testing, especially on the maximum capacity of the floating algal mass accommodating *Apohyale* and the controversial contribution of fragments on floating algal biomass (discussed below). As a primary consumer, *Apohyale* sp. consumed a substantial amount of primary production and also was preyed on by the marine macrobenthos, hence promoting energy flow through a trophic cascade. During the sampling cruise in June 2020, *Apohyale* sp. was frequently observed in the gastric contents of benthic Echinodermata captured by bottom trawling at Station Q (Figure 8). Large-scale field surveys on the macrofaunal community suggested an evident increase of Echinodermata abundance in the southern Yellow Sea in recent years (Xu et al., 2017), which indicated probably a fundamental ecological impact of the persisting YSGTs on the ecosystem of the Yellow Sea.

Another hypothesis associated with the grazing effects was that the algal fragments resulting from gnawing of animals could form sporangium which germinated *in situ* and caused an exponential increase of floating biomass (Gao et al., 2010; Wang G. C. et al., 2020). In this research, the *U. prolifera* fragments resulted from *Apohyale*'s gnawing maintained active photosynthesis. Extensive germination along with a higher biomass growth was observed in these fragments, indicating probable positive feedback on the floating biomass. In the laboratory, some fragments sunk to the bottom of beakers due to destroy of gas vesicles, the others stayed afloat. Both groups of fragments germinated and grew actively with unlimited illumination. In the natural ocean water, it is possible that floating fragments directly germinate and grow into drifting clumps under suitable environmental conditions. Whereas, further research is needed to understand the population dynamics of the sinking *U. prolifera* fragments in the field, such as deposition and recycling, under the complex food web in the southern Yellow Sea.

In summary, this research, for the first time, reported herbivorous gammarid species (*Apohyale* sp.) commonly inhabiting the floating mats of *U. prolifera*. Two grazing effects were revealed and quantitatively evaluated. The efficient grazing of *Apohyale* sp. consumed a substantial amount of floating biomass and dampened the rapid growth of *U. prolifera* in the eutrophied coastal seawater. At the same time, algal fragments resulting from gnawing might resume rapid growing and contribute positively to the floating biomass. The net gain or loss of floating biomass resulting from both effects probably needs to be studied in different blooming regions and at stages of YSGTs under variable grazing pressure and environmental conditions. Nonetheless, this research confirmed an important ecological function of the epiphytic herbivores on the biomass of YSGTs. More detailed studies are needed to elucidate the population dynamic of *Apohyale* sp. and the resulting biological roles on the floating *U. prolifera* green tides with the complex ecosystems in the southern Yellow Sea.

## Data Availability Statement

The original contributions presented in the study are included in the article/supplementary materials, further inquiries can be directed to the corresponding author/s.

## Author Contributions

XM designed the study, performed the research, analyzed the data, and wrote the manuscript. JX performed the research, analyzed the data, and prepared figures. SF and YZ collected samples. XZ and ZW contributed to the data analysis and revisions. All authors have reviewed, discussed, and agreed to the authorship and submission of the manuscript for peer review.

## Funding

This work was funded by the National Natural Science Foundation of China (41876137 and 41876199), the National Key Research and Development Program of China (2016YFC1402100), and NSFC-Shandong Joint Funded Project Marine Ecology and Environmental Sciences (U1606404).

## Conflict of Interest

The authors declare that the research was conducted in the absence of any commercial or financial relationships that could be construed as a potential conflict of interest.

## Publisher's Note

All claims expressed in this article are solely those of the authors and do not necessarily represent those of their affiliated organizations, or those of the publisher, the editors and the reviewers. Any product that may be evaluated in this article, or claim that may be made by its manufacturer, is not guaranteed or endorsed by the publisher.
