# Corrigendum: Assessing Herbivorous Impacts of *Apohyale* sp. on the *Ulva prolifera* Green Tide in China

**DOI:** 10.3389/fpls.2022.849440

**Published:** 2022-01-25

**Authors:** Xiaoxiang Miao, Jie Xiao, Shiliang Fan, Yu Zang, Xuelei Zhang, Zongling Wang

**Affiliations:** ^1^College of Environmental Science and Engineering, Ocean University of China, Qingdao, China; ^2^Key Laboratory of Marine Eco-Environmental Science and Technology, First Institute of Oceanography, Ministry of Natural Resources, Qingdao, China; ^3^Laboratory of Marine Ecology and Environmental Science, Pilot National Laboratory for Marine Science and Technology (Qingdao), Qingdao, China

**Keywords:** *Ulva prolifera*, *Apohyale* sp., grazing, green tides, Yellow Sea

In the original article, there was an error in the Title, Citation, Abstract, Figure 3 caption, and Discussion section. **The species name “*Apohale* sp.” was mistakenly spelled**.

A correction has been made to ***the Title***:

Assessing Herbivorous Impacts of *Apohyale* sp. on the *Ulva prolifera* Green Tide in China

A correction has been made to ***the Citation***:

Miao X, Xiao J, Fan S, Zang Y, Zhang X and Wang Z (2021) Assessing Herbivorous Impacts of *Apohyale* sp. on the *Ulva prolifera* Green Tide in China. *Front. Plant Sci.* 12:795560. 10.3389/fpls.2021.795560

A correction has been made to **the Abstract:**

It was estimated that grazing of *Apohyale* sp. could efficiently reduce ~0.4 and 16.6% of the algal growth rates in Rudong and Qingdao, respectively.

A correction has been made to **the Figure 3 caption:**

The condensed ML phylogenetic tree based on the amino acid sequences of COI in Hyalidae with cut-off value >30%. The monophyletic clade of each species was compressed and labeled with the specific markers and species names. Numbers above lines are bootstrapping support value (%) after 1,000 permutations. *Apohyale* sp. represents the sequence from this research. ML, Maximum-likelihood; COI, cytochrome oxidase I.

A correction has been made to **the Discussion:**

But the feasibility of this idealistic biological control method probably needs further testing, especially on the maximum capacity of the floating algalmass accommodating *Apohyale* and the controversial contribution of fragments on floating algal biomass (discussed below).

The authors apologize for this error and state that this does not change the scientific conclusions of the article in any way. The original article has been updated.

## Publisher's Note

All claims expressed in this article are solely those of the authors and do not necessarily represent those of their affiliated organizations, or those of the publisher, the editors and the reviewers. Any product that may be evaluated in this article, or claim that may be made by its manufacturer, is not guaranteed or endorsed by the publisher.

